# Supplementary material for: Functional Toll-Like Receptors (TLRs) Are Expressed by a Majority of Primary Human Acute Myeloid Leukemia Cells and Inducibility of the TLR Signaling Pathway Is Associated with a More Favorable Phenotype
Source: Cancers (Basel). 2019 Jul 11;11(7):973. doi: 10.3390/cancers11070973 (PMC6678780; doi:10.3390/cancers11070973)
Supplement: Supplementary file 1 [file cancers-11-00973-s001.pdf]

*Supplementary Material*

# Functional Toll-Like Receptors (TLRs) are Expressed by a Majority of Primary Human Acute Myeloid Leukemia Cells and Inducibility of the TLR Signaling Pathway is Associated with a More Favorable Phenotype

**Annette K. Brenner and Øystein Bruserud**

**Table S1.** Median concentration increase in the secretion of 19 (16 patients with G-CSF, 67 patients with GM-CSF) soluble mediators as a result of targeting with four different TLR agonists. More than 5-fold increase is highlighted.

| | LPS (TLR4) | Flagellin (TLR5) | R848 (TLR7/8) | Pam3CSK4 (TLR1/2) |
|---|---|---|---|---|
| IL-6 | **301.0** | **45.9** | **10.2** | **5.3** |
| TNFα | **188.7** | **10.6** | **5.0** | 1.5 |
| IL-1β | **89.7** | **9.2** | 1.1 | 1.5 |
| CCL3 | **56.4** | **6.0** | 2.4 | 1.5 |
| CCL2 | **52.6** | **9.4** | 4.3 | 2.8 |
| G-CSF | **51.9** | 2.7 | 1.0 | 1.0 |
| CXCL1 | **38.0** | **5.8** | 1.4 | 1.5 |
| CCL4 | **17.2** | 3.3 | 2.0 | 1.3 |
| CCL5 | **14.5** | 2.1 | 1.3 | 1.1 |
| MMP-1 | **11.1** | 3.4 | 1.4 | 1.1 |
| CXCL5 | **8.3** | 4.3 | 1.3 | 1.4 |
| CXCL8 | **6.0** | 4.3 | 1.7 | 1.3 |
| GM-CSF | **5.2** | 1.5 | 1.4 | 1.0 |
| MMP-2 | 4.0 | 1.5 | 1.2 | 1.1 |
| CXCL10 | 3.9 | 1.6 | 2.2 | 1.1 |
| HGF | 2.0 | 1.1 | 1.0 | 1.0 |
| MMP-9 | 1.9 | 2.5 | 1.3 | 1.7 |
| IL-1RA | 1.8 | 1.3 | 1.3 | 1.0 |
| Serpin E1 | 1.3 | 1.3 | 1.1 | 1.1 |
| Cystatin C | 1.1 | 1.1 | 1.0 | 1.0 |

**Table S2.** Detailed information about the 83 AML patients included in the study.

| ID | Gender | Age | Etiology | FAB | Cytogenetics | Mutations | CD34 | TLR1/2 responder | TLR responder |
|---|---|---|---|---|---|---|---|---|---|
| 1 | M | 42 | *de novo* | M2 | normal | *Flt3* | pos | partial | yes |
| 2 | F | 80 | MF | M2 | n.d. | n.d. | pos | no | yes |
| 3 | M | 79 | MDS | M2 | +8, -9 | wt | pos | no | yes |
| 4 | M | 60 | *de novo* | M5 | t(10;11), +8 | wt | n.d. | partial | yes |
| 5 | M | 33 | *de novo* | M4/5 | normal | *Flt3/NPM1* | neg | partial | yes |
| 6 | M | 78 | CMML | M4 | +8 | n.d. | neg | yes | yes |
| 7 | F | 49 | CML/relapse | M2 | complex | n.d. | pos | no | no |
| 8 | M | 33 | *de novo* | M2 | normal | wt | pos | partial | partial |
| 9 | F | 51 | *de novo* | M0 | complex | wt | pos | yes | yes |
| 10 | F | 67 | *de novo* | M5 | t(9;11), +19 | wt | neg | no | no |
| 11 | M | 71 | relapse | M4 | normal | wt | pos | partial | yes |
| 12 | M | 83 | *de novo* | M1 | n.d. | wt | pos | partial | yes |
| 13 | M | 24 | CMML | M2 | complex | n.d. | pos | no | no |
| 14 | F | 66 | *de novo* | M4/5 | t(9;15) | *Flt3* | neg | no | no |
| 15 | F | 77 | MDS | M1 | normal | wt | pos | partial | yes |
| 16_1 | M | 46 | *de novo* | M1 | normal | *NPM1* | n.d. | no | yes |
| 16_2 | M | 46 | relapse | M1 | normal | *NPM1* | n.d. | yes | yes |
| 17 | M | 68 | MF | M4 | normal | D835 | pos | no | no |
| 18 | F | 71 | MDS/relapse | n.d. | del(12) | n.d. | pos | yes | yes |
| 19 | F | 86 | *de novo* | M1 | normal | n.d. | pos | yes | yes |
| 20 | F | 70 | *de novo* | M4 | n.d. | *NPM1* | neg | yes | yes |
| 21 | M | 71 | *de novo* | M4/5 | n.d. | n.d. | n.d. | partial | yes |
| 22 | F | 18 | *de novo* | M4 | inv(16) | wt | pos | yes | yes |
| 23 | F | 77 | MDS | M1/2 | normal | *Flt3/NPM1* | n.d. | yes | yes |
| 24 | M | 71 | *de novo* | n.d. | n.d. | n.d. | n.d. | no | no |
| 25 | F | 46 | *de novo* | M1 | inv(16) | wt | pos | partial | yes |
| 26 | M | 65 | *de novo* | M5 | normal | *Flt3/NPM1* | neg | yes | yes |
| 27 | F | 75 | MF | M2 | n.d. | n.d. | pos | yes | yes |
| 28 | F | 78 | *de novo* | M1 | normal | *Flt3/NPM1* | neg | yes | yes |
| 29 | F | 42 | *de novo* | M5 | normal | *NPM1* | neg | yes | yes |
| 30 | F | 67 | *de novo* | M0 | +21 | wt | pos | no | no |

| | | | | | | | | | |
|---|---|---|---|---|---|---|---|---|---|
| 31 | M | 60 | MDS | n.d. | normal | n.d. | pos | no | no |
| 32 | M | 60 | *de novo* | M4 | del(9) | *Flt3* | pos | partial | yes |
| 33 | F | 49 | *de novo* | M5 | normal | *Flt3/NPM1* | neg | yes | yes |
| 34 | M | 41 | *de novo* | M1 | t(8;21), del(9), -20, -22 | wt | pos | no | no |
| 35 | M | 35 | *de novo* | M2 | normal | wt | pos | no | yes |
| 36 | M | 76 | MDS | n.d. | normal | n.d. | pos | partial | yes |
| 37 | M | 72 | MDS | M1 | complex | wt | pos | no | yes |
| 38 | F | 71 | MDS | n.d. | t(1;5), t(2;3) | n.d. | pos | yes | yes |
| 39 | F | 75 | *de novo* | M4 | normal | *Flt3* | pos | yes | yes |
| 40 | F | 74 | CMML | M5 | normal | *Flt3/NPM1* | neg | partial | yes |
| 41 | M | 79 | *de novo* | M1 | n.d. | wt | pos | n.d. | no |
| 42 | M | 82 | *de novo* | n.d. | +8 | wt | pos | yes | yes |
| 43 | M | 58 | *de novo* | M5 | normal | wt | pos | no | partial |
| 44 | F | 55 | *de novo* | M5 | normal | *Flt3/NPM1* | neg | yes | yes |
| 45 | M | 65 | MDS | M4/5 | normal | *NPM1* | neg | yes | yes |
| 46 | M | 72 | *de novo* | M5 | normal | *NPM1* | neg | no | yes |
| 47 | M | 64 | *de novo* | M5 | normal | *NPM1* | neg | partial | yes |
| 48 | M | 36 | *de novo* | M5 | +8, +22, inv(16) | *Flt3* | pos | no | partial |
| 49_1 | M | 48 | *de novo* | M5 | normal | *Flt3/NPM1* | n.d. | yes | yes |
| 49_2 | M | 48 | relapse | M5 | normal | *Flt3/NPM1* | n.d. | no | no |
| 50 | M | 78 | *de novo* | M1 | complex | n.d. | pos | no | no |
| 51 | M | 36 | *de novo* | M4 | inv(16) | wt | pos | partial | yes |
| 52 | F | 92 | CLL | M1 | n.d. | n.d. | neg | no | no |
| 53 | F | 59 | chemo | M4 | normal | *Flt3/NPM1* | neg | n.d. | yes |
| 54_1 | M | 61 | *de novo* | M4 | normal | wt | neg | n.d. | yes |
| 54_2 | M | 61 | relapse | M4 | normal | wt | neg | no | no |
| 55 | M | 73 | *de novo* | M2 | del(7), -20 | *Flt3* | pos | partial | partial |
| 56 | F | 57 | *de novo* | M4 | inv(16) | wt | pos | partial | yes |
| 57 | M | 20 | *de novo* | M2 | normal | *Flt3* | pos | yes | yes |
| 58 | F | 64 | *de novo* | M2 | normal | *Flt3/NPM1* | neg | no | no |
| 59 | M | 87 | MF | M1 | del(20) | wt | pos | no | partial |
| 60 | F | 29 | *de novo* | M5 | normal | *Flt3*/D835 | pos | yes | yes |
| 61 | F | 61 | MDS/relapse | M1 | complex | wt | neg | partial | yes |

| | | | | | | | | | |
|---|---|---|---|---|---|---|---|---|---|
| 62 | F | 80 | PV | M4/5 | complex | wt | n.d. | partial | yes |
| 63 | F | 74 | *de novo* | M4 | t(8;21) | *Flt3* | pos | yes | yes |
| 64 | M | 74 | *de novo* | M1 | complex | wt | pos | n.d. | no |
| 65 | M | 78 | *de novo* | M0 | complex | wt | neg | no | no |
| 66 | M | 65 | *de novo* | M5 | complex | *NPM1* | n.d. | yes | yes |
| 67 | F | 64 | relapse | M4 | complex | wt | pos | partial | yes |
| 68 | M | 67 | relapse | M1 | normal | *Flt3* | pos | partial | partial |
| 69 | M | 64 | relapse | M2 | normal | *NPM1* | neg | yes | yes |
| 70 | F | 81 | MDS | M1 | normal | wt | pos | partial | yes |
| 71 | M | 72 | MDS | M4 | normal | n.d. | neg | no | yes |
| 72 | F | 75 | *de novo* | M1 | n.d. | *Flt3* | pos | no | partial |
| 73 | M | 77 | *de novo* | M2 | complex | *Flt3* | pos | no | yes |
| 74 | F | 60 | *de novo* | M5 | normal | *Flt3/NPM1* | pos | yes | yes |
| 75 | F | 55 | *de novo* | M1 | normal | *Flt3/NPM1* | pos | no | yes |
| 76 | M | 62 | *de novo* | M4 | +8 | wt | pos | n.d. | yes |
| 77 | F | 87 | *de novo* | M0 | del(5) | wt | pos | partial | yes |
| 78 | M | 59 | CMML/relapse | M5 | del(20), +8 | *Flt3* | pos | no | partial |
| 79 | F | 71 | *de novo* | M0 | normal | *NPM1* | neg | yes | yes |
| 80 | F | 46 | *de novo* | M2 | inv(13) | wt | pos | no | partial |
| 81 | F | 63 | *de novo* | M4 | normal | *Flt3* | pos | no | partial |
| 82 | F | 77 | *de novo* | M1 | n.d. | *NPM1* | neg | no | yes |
| 83 | M | 19 | *de novo* | M5 | normal | wt | neg | yes | yes |

| | Legend to color codes |
|---|---|
| (green) | Survival data |
| (blue) | Microarray |
| (orange) | Survival data and microarray |

**Table S3.** GO-terms and their associated proteins that were found to be upregulated in TLR non-responders and TLR responders, respectively. Color-highlighted proteins were identified both by functional annotation and gene classification in DAVID 6.8. All information was obtained from genecards.org.

| **Upregulated in TLR non-responders** | |
|---|---|
| **GO-term: 0006351 (transcription, DNA-templated), enrichment score: 4.89, *p*-value: 1.8 × 10$^{-7}$** | |
| **Gene names** | **Description** |
| ARID2 | **AT-rich interaction domain 2.** Members of the ARID family have roles in embryonic patterning, cell lineage gene regulation, cell cycle control, transcriptional regulation and chromatin structure modification. This protein functions as a subunit of the polybromo- and BRG1-associated factor or **SWI/SNF** chromatin remodeling complex which facilitates ligand-dependent transcriptional activation by nuclear receptors. |
| ASXL1 | **Additional sex combs like 1, transcriptional regulator.** The protein is a member of the **polycomb** group of proteins, which are necessary for the maintenance of stable repression of homeotic and other loci. The protein is thought to disrupt chromatin in localized areas, enhancing transcription of certain genes while repressing the transcription of other genes. |
| BMI1 | **BMI1 proto-oncogene, polycomb ring finger.** A major component of the **polycomb** group complex 1, which functions through chromatin remodeling as an essential epigenetic repressor of multiple regulatory genes involved in embryonic development and self-renewal in somatic stem cells. This protein also plays a central role in DNA damage repair. This gene is an **oncogene** and aberrant expression is associated with numerous cancers and is associated with resistance to certain chemotherapies. Read-through transcription also exists between this gene and the upstream COMMD3 gene (see below). |
| BRWD1 | **Bromodomain and WD repeat domain containing 1.** This gene encodes a member of the WD repeat protein family, which are involved in a variety of cellular processes including cell cycle progression, signal transduction, apoptosis, and gene regulation. This protein contains 2 bromodomains and multiple WD repeats. By similarity, the nuclear protein functions as a transcriptional activation domain which may regulate chromatin remodeling and associates with a component of the **SWI/SNF** chromatin remodeling complex. |
| CBX2 | **Chromobox 2.** This gene encodes a component of the **polycomb** multiprotein complex, which is required to maintain the transcriptionally repressive state of many genes throughout development via chromatin remodeling and modification of histones. |
| CHD8 | **Chromodomain helicase DNA binding protein 8.** This gene encodes a member of the chromodomain-helicase-DNA binding protein family, which is characterized by a **SNF2-like** domain and two chromatin organization modifier domains. This gene has been shown to function in several processes that include transcriptional regulation, epigenetic remodeling, promotion of cell proliferation, and regulation of RNA synthesis. |
| CHD9 | **Chromodomain helicase DNA binding protein 9.** Acts as a transcriptional coactivator for PPARA and possibly other nuclear receptors. Proposed to be a ATP-dependent chromatin remodeling protein. Has DNA-dependent ATPase activity and binds to A/T-rich DNA. |
| COMMD3 | **COMM domain containing 3.** May modulate activity of cullin-RING E3 ubiquitin ligase complexes. **May down-regulate activation of NFκB.** |
| CXXC5 | **CXXC finger protein 5.** The protein encoded by this gene is a retinoid-inducible nuclear protein containing a CXXC-type zinc finger motif. The encoded protein is involved in myelopoiesis and is required for DNA damage-induced p53 activation. |
| ELF2 | **E74 like ETS transcription factor 2.** Isoform 1 transcriptionally activates the LYN and BLK promoters and acts synergistically with RUNX1 to transactivate the BLK promoter. Isoform 2 may function in repression of RUNX1-mediated transactivation. |
| EZH2 | **Enhancer of zeste 2 polycomb repressive complex 2 subunit.** This gene encodes a member of the **polycomb**-group family, which are involved in maintaining the transcriptional repressive state of genes over successive cell generations. This protein may play a role in **hematopoiesis**. |
| FOXJ3 | **Forkhead box J3.** Transcriptional activator of MEF2C involved in the regulation pf adult muscle fiber type identity and skeletal muscle regeneration. |
| ILF3 | **Interleukin enhancer binding factor 3.** This gene encodes a dsRNA binding protein that complexes with other proteins, dsRNAs, small noncoding RNAs, and mRNAs to regulate gene expression and stabilize mRNAs. This protein forms a heterodimer with a 45 kDa transcription factor ILF2 required for T-cell expression of interleukin 2. This complex has been shown to affect the redistribution of nuclear mRNA to the cytoplasm. |
| MAGEL2 | **MAGE family member L2.** Probably enhances ubiquitin ligase activity of RING-type zinc finger-containing E3 ubiquitin-protein ligases, possibly through recruitment and/or stabilization of the Ubl-conjugating enzyme (E2) at the E3 substrate complex. |

| | |
|---|---|
| MAP3K7 | **Mitogen-activated protein kinase kinase kinase 7**. The protein encoded by this gene is a member of the serine/threonine protein kinase family. This kinase mediates the signaling transduction induced by TGFβ and morphogenetic protein (BMP) and controls a variety of cell functions including transcription regulation and apoptosis. In response to IL-1, this protein forms a kinase complex including TRAF6, MAP3K7P1/TAB1 and MAP3K7P2/TAB2, which **is required for the activation of NFκB.** |
| MZF1 | **Myeloid zinc finger 1.** Binds to target promoter DNA and functions as transcription regulator. May be one regulator of transcriptional events during **hematopoietic development**. |
| POLR1D | **RNA polymerase I subunit D.** The protein encoded by this gene is a component of the RNA polymerase I and RNA polymerase III complexes, which function in the synthesis of ribosomal RNA precursors and small RNAs, respectively. |
| PSIP1 | **PC4 and SFRS1 interacting protein 1.** Transcriptional coactivator involved in neuroepithelial stem cell differentiation and neurogenesis. May play a protective role during stress-induced apoptosis. Isoform 2 is a more general and stronger transcriptional coactivator and may also act as an adapter to coordinate pre-mRNA splicing. |
| RBBP4 | **RB binding protein 4, chromatin remodeling factor.** This gene encodes a ubiquitously expressed nuclear protein which belongs to a highly conserved subfamily of WD-repeat proteins. It is present in protein complexes involved in histone acetylation and chromatin assembly. It is part of the Mi-2 complex which has been implicated in chromatin remodeling and transcriptional repression associated with histone deacetylation. This encoded protein is also part of co-repressor complexes, which is an integral component of transcriptional silencing. It is found among several cellular proteins that bind directly to retinoblastoma protein to regulate cell proliferation. It also seems to be involved in transcriptional repression of E2F-responsive genes. |
| RCOR3 | **REST corepressor 3.** May act as a component of a corepressor complex that represses transcription. |
| SAFB2 | **Scaffold attachment factor B2.** The protein encoded by this gene, along with its paralog SAFB1, is a repressor of estrogen receptor $\alpha$. The encoded protein binds scaffold/matrix attachment region DNA and is involved in cell cycle regulation, apoptosis, differentiation, stress response, and regulation of immune genes. |
| SCMH1 | **Sex comb on midleg homolog 1.** Associates with **polycomb** group multiprotein complexes, which is required to maintain the transcriptionally repressive state of some genes. |
| SETD1A | **SET domain containing 1A.** The protein encoded by this gene is a component of a histone methyltransferase complex that produces mono-, di-, and tri-methylated histone H3 at Lys4. Tri-methylation of histone H3 at lysine 4 (H3K4me3) is a chromatin modification known to generally mark the transcription start sites of active genes. |
| SMARCC1 | **SWI/SNF related, matrix associated, actin dependent regulator of chromatin subfamily c member 1.** The protein encoded by this gene is a member of the **SWI/SNF** family of proteins, whose members display helicase and ATPase activities and which are thought to regulate transcription of certain genes by altering the chromatin structure around those genes. The encoded protein is part of the large ATP-dependent chromatin remodeling complex SNF/SWI and contains a predicted leucine zipper motif typical of many transcription factors. |
| TCF3 | **Transcription factor 3.** This gene encodes a member of the E protein (class I) family of helix-loop-helix transcription factors. E proteins activate transcription by binding to regulatory E-box sequences on target genes as heterodimers or homodimers and are inhibited by heterodimerization with inhibitor of DNA-binding (class IV) helix-loop-helix proteins. E proteins play a critical role in lymphopoiesis, and the encoded protein is required for B and T lymphocyte development. Deletion of this gene or diminished activity of the encoded protein may play a role in lymphoid malignancies. |
| TP53INP1 | **Tumor protein p53 inducible nuclear protein 1.** Antiproliferative and proapoptotic protein involved in cell stress response which acts as a dual regulator of transcription and autophagy. Acts as a positive regulator of autophagy. Acts as an antioxidant and plays a major role in p53/TP53-driven oxidative stress response. Possesses both a p53/TP53-independent intracellular reactive oxygen species regulatory function and a p53/TP53-dependent transcription regulatory function. Positively regulates p53/TP53 and p73/TP73 and stimulates their capacity to induce apoptosis and regulate cell cycle. In response to ds DNA breaks, promotes p53/TP53 phosphorylation on Ser-46 and subsequent apoptosis. Acts as a tumour suppressor by inducing cell death by an autophagy and caspase-dependent mechanism. Can reduce cell migration by regulating the expression of SPARC. |
| TRIM33 | **Tripartite motif containing 33.** Acts as an E3 ubiquitin-protein ligase. Promotes SMAD4 ubiquitination, nuclear exclusion and degradation via the ubiquitin proteasome pathway. May act as a transcriptional repressor. Inhibits the transcriptional response to TGFβ/BMP signaling cascade. Plays a role in the control of cell proliferation. By similarity, its association with SMAD2 and SMAD3 stimulates **erythroid differentiation** of hematopoietic stem/progenitor cells. |
| ZBTB20 | **Zinc finger and BTB domain containing 20.** May be a transcription factor involved in **hematopoiesis**, **oncogenesis**, and immune response. |
| ZNF219 | **Zinc finger protein 219.** This gene is a member of the Krüppel-like zinc finger gene family. The encoded protein functions as a transcriptional repressor of the high mobility group nucleosome binding domain 1 protein, which is associated with transcriptionally active chromatin. |
| ZNF251 | **Zinc finger protein 251**. May be involved in transcriptional regulation. |
| ZNF395 | **Zinc finger protein 395.** Plays a role in papillomavirus genes transcription. |

| | |
|---|---|
| ZNF431 | **Zinc finger protein 431.** This gene encodes a member of the Krüppel C2H2-type zinc-finger family of proteins. The encoded protein may negatively regulate transcription of target genes, including the hedgehog signaling pathway receptor patched 1 by interacting with histone deacetylases. |
| ZNF521 | **Zinc finger protein 521.** Transcription factor that can both act as an activator or a repressor depending on the context. Involved in BMP signaling and in the regulation of the immature compartment of the hematopoietic system. Associates with SMADs in response to BMP2 leading to activate transcription of BMP target genes. Acts as a transcriptional repressor via its interaction with EBF1, a transcription factor involved specification of B-cell lineage; this interaction preventing EBF1 to bind DNA and activate target genes. |
| ZNF548 | **Zinc finger protein 548.** May be involved in transcriptional regulation. |
| ZNF669 | **Zinc finger protein 669.** May be involved in transcriptional regulation. |
| ZNF700 | **Zinc finger protein 700.** May be involved in transcriptional regulation. |
| ZNF763 | **Zinc finger protein 763.** May be involved in transcriptional regulation. |
| ZNF789 | **Zinc finger protein 789.** May be involved in transcriptional regulation. |
| GO-term 0006357: regulation of transcription from RNA polymerase II promoter, $p = 1.8 \times 10^{-5}$ | |
| RFX7 | **Regulatory factor X7.** A member of the RFX family of transcription factors. |
| GO-term 0006351: transcription, DNA-templated; $p = 6.0 \times 10^{-12}$ | |
| InterPro-term 001680: WD40-repeat, $p = 4.3 \times 10^{-8}$ | |
| FBXW9 | **F-box and WD repeat domain containing 9.** SCF complexes, formed by SKP1, cullin and F-box proteins, act as protein-ubiquitin ligases. F-box proteins interact with SKP1 through the F-box, and they interact with ubiquitination targets through other protein interaction domains |
| PHIP | **Pleckstrin homology domain interacting protein.** Probable regulator of the insulin and insulin-like growth factor signalling pathways. Stimulates cell proliferation through regulation of cyclin transcription and has an anti-apoptotic activity through AKT1 phosphorylation and activation. Plays a role in the regulation of cell morphology and cytoskeletal organization. |
| WDR33 | **WD repeat domain 33.** Members of this family are involved in a variety of cellular processes, including cell cycle progression, signal transduction, apoptosis, and gene regulation. Essential for both cleavage and polyadenylation of pre-mRNA 3 ends. |

| **Upregulated in TLR responders** | |
|---|---|
| **GO-term: 0071222 (cellular response to LPS), enrichment score: 0.07, *p*-value: 2.6 × 10$^{-3}$** | |
| **Gene names** | **Description** |
| CD86 | **CD86 molecule.** This gene encodes a type I membrane protein that is a member of the immunoglobulin superfamily. It is expressed by antigen-presenting cells, and it is the ligand for two proteins at the cell surface of T cells, CD28 antigen and cytotoxic T-lymphocyte-associated protein 4. Binding of this protein with CD28 antigen is a costimulatory signal for activation of the T-cell. Binding of this protein with cytotoxic T-lymphocyte-associated protein 4 negatively regulates T-cell activation and diminishes the immune response. |
| CXCL16 | **C-X-C motif chemokine ligand 16.** By similarity, the encoded protein acts as a scavenger receptor on macrophages and specifically binds to oxidized LDL, suggesting that it may be involved in pathophysiology such as atherogenesis. Induces a strong chemotactic response and calcium mobilization. |
| HAVCR2 | **Hepatitis A virus cellular receptor 2.** The protein encoded by this gene belongs to the immunoglobulin superfamily, and TIM family of proteins. This protein is a Th1-specific cell surface protein that regulates macrophage activation and inhibits Th1-mediated auto- and allo-immune responses, and promotes immunological tolerance. Expressed on dendritic cells, it positively regulates innate immune response and in synergy with TLRs promotes secretion of TNF-α. |
| TNFRSF1B | **TNF receptor superfamily member 1B.** Receptor with high affinity for TNFSF2/TNFα and approximately 5-fold lower affinity for homotrimeric TNFSF1/lymphotoxin-α. The TRAF1/TRAF2 complex recruits the apoptotic suppressors BIRC2 and BIRC3 to TNFRSF1B/TNFR2. This receptor mediates most of the metabolic effects of TNF-α. Isoform 2 blocks TNFα-induced apoptosis, which suggests that it regulates TNFα function by antagonizing its biological activity. |

**Table S4.** Overview over the mutational landscape of 38 consecutive AML patients. TLR responders and non-responders do not differ in any of the 37 assessed genes.

| | | **TLR responders** | | |
|---|---|---|---|---|
| | | **Yes (n=31)** | **No (n=7)** | ***p*-value** |
| Signaling | *Flt3*-ITD | 5 (16%) | 2 (29%) | 1.000 |
| | *Flt3*-TKD | 3 (10%) | 2 (29%) | 0.223 |
| | *HRAS* | 1 (3%) | 0 | 1.000 |
| | *JAK2* | 1 (3%) | 0 | 1.000 |
| | *KIT* | 1 (3%) | 0 | 1.000 |
| | *KRAS* | 3 (10%) | 1 (14%) | 1.000 |
| | *NOTCH1* | 1 (3%) | 0 | 1.000 |
| | *NRAS* | 7 (23%) | 0 | 0.309 |
| | *PTPN11* | 3 (10%) | 0 | 1.000 |
| | Total group | 25 mutations | 5 mutations | 1.000 |
| Tumor suppressors | *CDKN2A* | 1 (3%) | 0 | 1.000 |
| | *CUX1* | 0 | 0 | - |
| | *IKZF1* | 3 (10%) | 1 (14%) | 1.000 |
| | *PHF6* | 0 | 0 | 1.000 |
| | *TP53* | 2 (6%) | 1 (14%) | 0.467 |
| | *WT1* | 2 (6%) | 0 | 1.000 |
| | Total group | 6 mutations | 2 mutations | 0.642 |
| Epigenetic modifiers | *ASXL1* | 6 (19%) | 1 (14%) | 1.000 |
| | *DNMT3A* | 11 (35%) | 1 (14%) | 0.395 |
| | *EZH2* | 2 (6%) | 1 (14%) | 0.467 |
| | *IDH1* | 1 (3%) | 1 (14%) | 0.339 |
| | *IDH2* | 2 (6%) | 2 (29%) | 0.147 |
| | *KDM6A* | 0 | 0 | - |
| | *KMT2A/MLL* | 0 | 0 | - |
| | *TET2* | 7 (23%) | 0 | 0.309 |
| | Total group | 29 mutations | 6 mutations | 1.000 |
| Myeloid TFs | *CEBPA* | 5 (16%) | 1 (14%) | 1.000 |
| | *GATA2* | 3 (10%) | 0 | 1.000 |
| | *RUNX1* | 7 (23%) | 0 | 0.309 |
| | Total group | 15 mutations | 1 mutation | 0.297 |
| Spliceosome/ transcription repressors | *BCOR* | 1 (3%) | 1 (14%) | 0.339 |
| | *BCORL1* | 1 (3%) | 0 | 1.000 |
| | *SF3B1* | 2 (6%) | 0 | 1.000 |
| | *SRSF2* | 3 (10%) | 1 (14%) | 1.000 |
| | *ZRSR2* | 1 (3%) | 0 | 1.000 |
| | Total group | 8 mutations | 2 mutations | 1.000 |
| Cohesin | *RAD21* | 1 (3%) | 0 | 1.000 |
| | *SMC1A* | 0 | 1 (14%) | 0.184 |
| | *STAG2* | 3 (10%) | 0 | 1.000 |
| | Total group | 4 mutations | 1 mutation | 1.000 |
| Others | *CSF3R* | 1 (3%) | 0 | 1.000 |
| | *NPM1*-ins | 5 (16%) | 2 (29%) | 1.000 |
| | *SETBP1* | 0 | 1 (14%) | 0.184 |
| | Total | 95 mutations | 20 mutations | 0.889 |
| | Per patient | 3.1 mutations | 2.9 mutations | |

**Table S5.** Overexpressed genes in TLR non-responders and responders that can be linked with AML prognosis.

| **Overexpressed in TLR non-responders** | | | |
|---|---|---|---|
| **Protein name** | **Impact in AML** | **Prognosis** | Reference |
| AF1Q/ MLLT11 | Associated with higher blast count and adverse prognosis. | Adverse | [1,2] |
| BARD1/ FLJ22536 | Downregulated when MDS progresses to AML. | Favorable? | [3] |
| BMI1 | Increased in patients with adverse prognosis. High levels favor proliferation over cell cycle arrest. Associated with higher blast burden. | Adverse | [4,5] |
| CBX2 | Regulates tumorigenicity. Overexpression is associated with shorter survival in several tumors. | Adverse | [6] |
| CUL9 | p53 inhibitor. Associated with *Flt3*-ITD. May be essential for differentiation block in AML. | Adverse | [7] |
| CXX5 | Overexpression is associated with adverse prognosis, while low levels are linked with lower relapse rate, pro-longed survival and downregulation of genes involved in leukemogenesis. | Adverse | [8–10] |
| DACH1 | Downregulated during myeloid differentiation. | Adverse? | [11] |
| EZH2 | High mRNA expression is associated with high blood blast number, extramedullary infiltration and shorter time to relapse. | Adverse | [12] |
| MAP3K7 | Knockdown lead to impaired leukemia development in mice and longer survival. Is associated with adverse prognosis in other types of cancer. | Adverse | [13] |
| MYL6B | mRNA overexpression is associated with poor prognosis in liver cancer. Promotes p53 degradation. | Adverse? | [14] |
| NIPA1/ SP6G | High expression is inversely correlated with patient survival. Supports growth and survival of blasts. | Adverse | [15] |
| OTUD5 | Destabilizes p53 by ubiquitination. | Adverse? | [16] |
| PRDX2 | Reduces the intracellular levels of reactive oxygen species and is regarded a tumor suppressor. Is also linked with mutations in *DNMT3A*, apoptosis block and enhanced blast proliferation. | Favorable?/ Adverse? | [17,18] |
| RCOR3 | Inhibits hematopoietic differentiation. | Adverse | [19] |
| SETD1A | Important for AML proliferation and blast survival. | Adverse | [20] |
| SIAH1 | Proteasomal degradation of *Flt3*-ITD. | Favorable? | [21] |
| TCF3 | Related to leukemia development and progression. | Adverse | [22] |
| TP53INP1 | Tumor suppressor. High expression is linked with pro-longed survival after stem cell transplantation. | Favorable | [23] |
| TSPAN3 | Necessary for development and progression of AML. | Adverse | [24] |
| ZBTB20 | Important for myeloid development. Necessary to promote full TLR activation. | Favorable | [25] |

| **Overexpressed in TLR responders** | | | |
|---|---|---|---|
| **Protein name** | **Impact in AML** | **Prognosis** | **Reference** |
| CASP1 | Associated with expression of pro-inflammatory cytokines via NFκB. Correlates with high expression of S100A8 and A9. | Neutral? | [26] |
| CD31/ PECAM1 | Correlated with poor prognosis. Promotes tumor progression through Akt signaling. | Adverse | [27] |
| CD86 | Myeloid differentiation marker. Associated with FAB M4/M5. | Favorable | [28] |
| FTL | Associated with *NPM1*-insertions. | Favorable? | [29] |
| HAVCR2/ TIM-3 | Associated with FAB M4, inv(16) and complete remission. Absent on normal hematopoietic cells. | Favorable | [30,31] |
| ILK | Pro-oncogene; overexpression is linked with increased blast proliferation. Is also a TLR4 regulator, and a regulator of expression of pro-inflammatory cytokines. | Adverse | [32,33] |
| LYZ | Usually underexpressed in AML. Expression is associated with more differentiated cells. | Favorable? | [34] |
| MAPK1A | Contributes to AML. | Adverse | [35] |
| MLKL | Especially low expression in *Flt3*-ITD. | Favorable? | [36] |
| TNFAIP8 | Higher expression is correlated with complete remission as opposed to blast persistence. | Favorable | [37] |
| YWHAB | High expression may lead to oncogenesis by upregulating MDM2 – a p53 negative regulator. | Adverse | [38] |

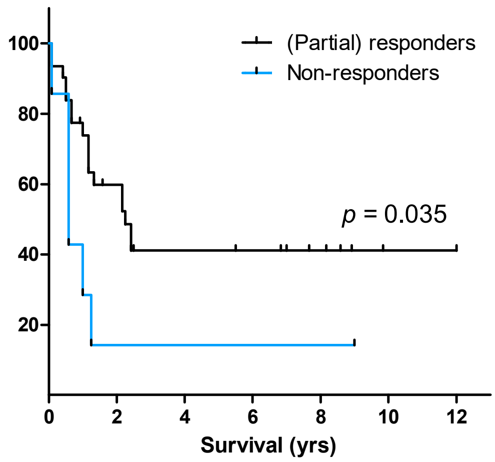


**Figure S1.** Patient survival dependent on response towards general TLR targeting. Lack of mediator secretion upon general TLR targeting is associated with an adverse prognosis (log-rank test) but is not an independent prognostic factor. Seven patients were categorized as non-responders and 31 as responders, respectively.

| Variable | Adj. HR | 95% CI | *p*-value |
|---|---|---|---|
| TLR1 non-responder [1] | 1.47 | 0.74 – 2.93 | 0.275 |
| Age (≥60 years) | 1.38 | 0.77 – 2.49 | 0.285 |
| Etiology (secondary) | 3.24 | 0.74 – 14.26 | 0.120 |
| Cytogenetics (intermediate/ adverse) | 0.39 | 0.10 – 1.58 | 0.187 |
| *Flt3*-ITD | 2.55 | 0.84 – 7.76 | 0.099 |
| *NPM1*-insertion | 1.01 | 0.29 – 3.55 | 0.986 |

[1] Reference value is indicated.

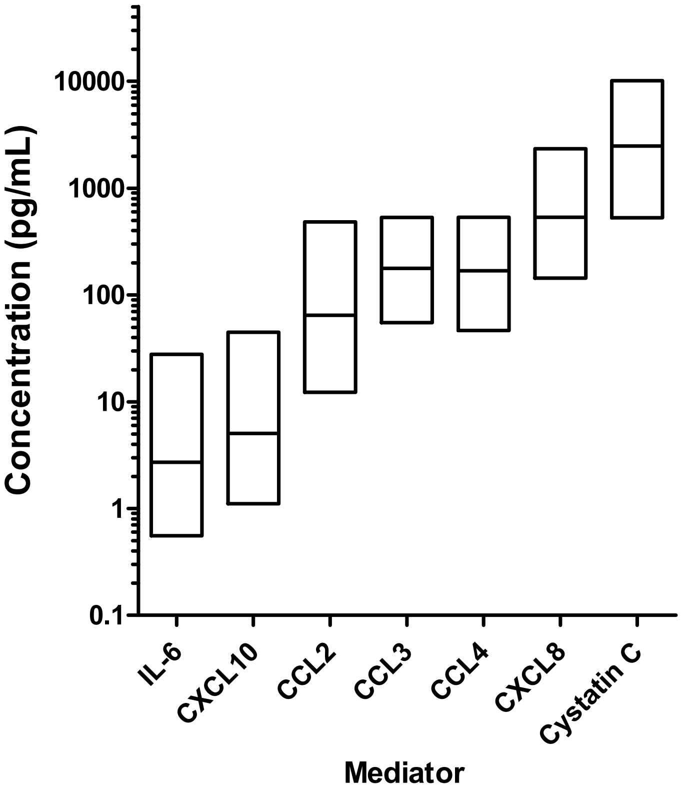


| | IL-6 | CXCL10 | CCL2 | CCL3 | CCL4 | CXCL8 | Cystatin C |
|---|---|---|---|---|---|---|---|
| Below detection limit | 19 | 25 | 13 | 20 | 33 | 0 | 14 |
| Above detection limit | 1 | 4 | 0 | 0 | 0 | 14 | 0 |

**Figure S2.** Basal mediator secretion levels for the 83 AML patients. Boxplot showing the 25$^{th}$ and 75$^{th}$ quartiles in addition to the median of the seven soluble mediators with the highest basal concentration ranges among the 83 patients. These large differences are unlikely to be caused by the minority of contaminating normal lymphocytes and monocytes in the samples. The table further provides an overview of the number of samples, where concentrations were outside the extrapolated standard curve of the Luminex assays. These values were not included in the figure for clarity.

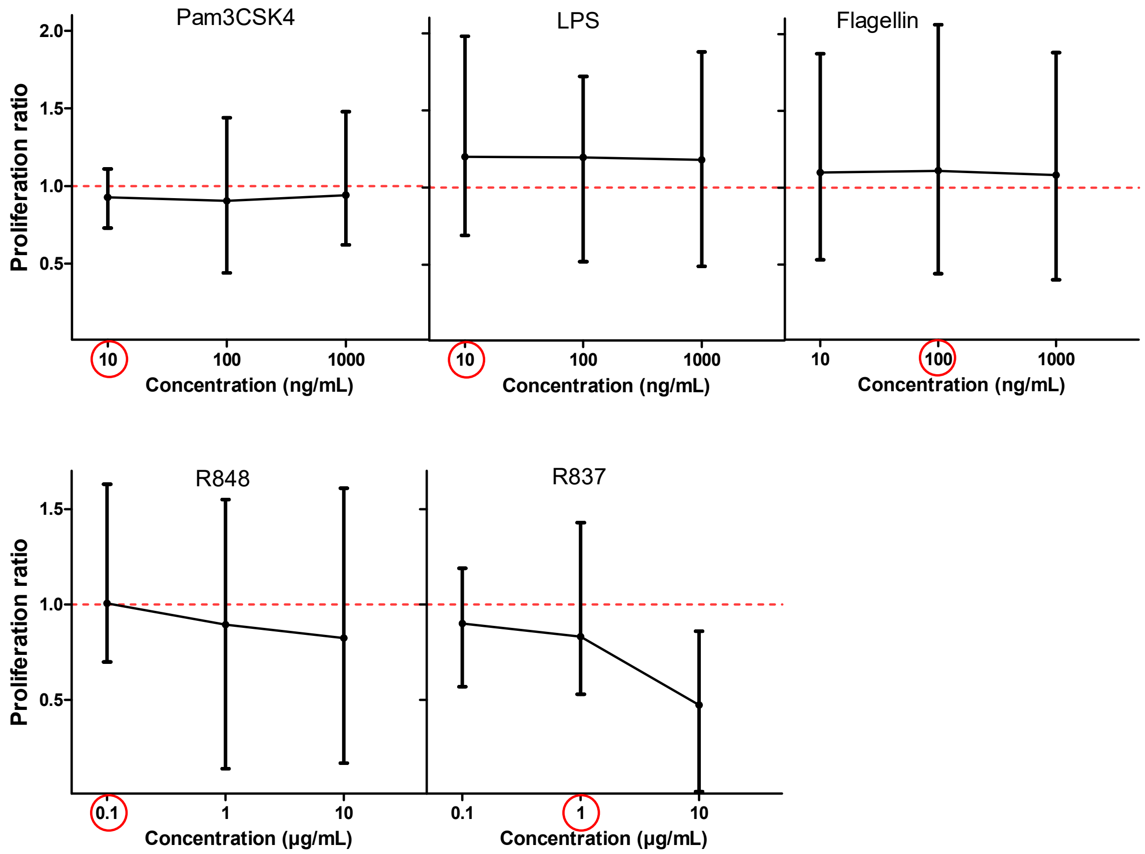


**Figure S3.** Change in proliferation (total range is shown) for the 10 test patients whose cells were exposed to different concentrations of TLR ligands. Only high doses of the TLR7 agonist R837 (imiquimod) reduced proliferation capability of the AML cells in accordance with a previous study on 10 AML cell lines [39]. The chosen concentrations in the extended study on 83 patients are highlighted. R837 at 1 µg/mL only resulted in significant elevated cytokine secretion (we tested for CCL2, CCL3, CCL4, CXCL8 and IL-6) for two patients; therefore, we excluded the agonist from further experiments. .

## Proliferation method

Patient cells (5.0 × $10^4$ cells/well) were cultured for two days in StemSpan medium in presence of 20 ng/mL of the haematopoietic growth factors GM-CSF, Flt3ligand and stem cell factor. 37 kBq of $^3$H-thymidine (Perkin Elmer; Walthman, MA, USA) were added to each well on day 2 and the cells cultured for an additional day, before nuclear thymidine incorporation was assessed. Change in proliferation was calculated as proliferation ratio compared to the untreated control.

## References

1. Akhter, A.; Farooq, F.; Elyamany, G.; Mughal, M.K.; Rashid-Kolvear, F.; Shabani-Rad, M.T.; Street, L.; Mansoor, A. Acute Myeloid Leukemia (AML): Upregulation of BAALC/MN1/MLLT11/EVI1 Gene Cluster Relate With Poor Overall Survival and a Possible Linkage With Coexpression of MYC/BCL2 Proteins. *Appl. Immunohistochem. Mol. Morphol.* **2018**, *26*, 483–488, doi:10.1097/pai.0000000000000452.
2. Strunk, C.J.; Platzbecker, U.; Thiede, C.; Schaich, M.; Illmer, T.; Kang, Z.; Leahy, P.; Li, C.; Xie, X.; Laughlin, M.J.; et al. Elevated AF1q expression is a poor prognostic marker for adult acute myeloid leukemia patients with normal cytogenetics. *Am. J. Hematol.* **2009**, *84*, 308–309, doi:10.1002/ajh.21396.
3. Mahadevan, D.; DiMento, J.; Croce, K.D.; Riley, C.; George, B.; Fuchs, D.; Mathews, T.; Wilson, C.; Lobell, M. Transcriptosome and serum cytokine profiling of an atypical case of myelodysplastic syndrome with progression to acute myelogenous leukemia. *Am. J. Hematol.* **2006**, *81*, 779–786, doi:10.1002/ajh.20690.

4. Darwish, N.H.; Sudha, T.; Godugu, K.; Elbaz, O.; Abdelghaffar, H.A.; Hassan, E.E.; Mousa, S.A. Acute myeloid leukemia stem cell markers in prognosis and targeted therapy: Potential impact of BMI-1, TIM-3 and CLL-1. *Oncotarget* **2016**, *7*, 57811–57820, doi:10.18632/oncotarget.11063.
5. Saudy, N.S.; Fawzy, I.M.; Azmy, E.; Goda, E.F.; Eneen, A.; Abdul Salam, E.M. BMI1 gene expression in myeloid leukemias and its impact on prognosis. *Blood Cells Mol. Dis.* **2014**, *53*, 194–198, doi:10.1016/j.bcmd.2014.07.002.
6. Di Costanzo, A.; Del Gaudio, N.; Conte, L.; Dell'Aversana, C.; Vermeulen, M.; de The, H.; Migliaccio, A.; Nebbioso, A.; Altucci, L. The HDAC inhibitor SAHA regulates CBX2 stability via a SUMO-triggered ubiquitin-mediated pathway in leukemia. *Oncogene* **2018**, *37*, 2559–2572, doi:10.1038/s41388-018-0143-1.
7. Seipel, K.; Marques, M.T.; Bozzini, M.A.; Meinken, C.; Mueller, B.U.; Pabst, T. Inactivation of the p53-KLF4-CEBPA Axis in Acute Myeloid Leukemia. *Clin. Cancer Res.* **2016**, *22*, 746–756, doi:10.1158/1078-0432.Ccr-15-1054.
8. Astori, A.; Fredly, H.; Aloysius, T.A.; Bullinger, L.; Mansat-De Mas, V.; de la Grange, P.; Delhommeau, F.; Hagen, K.M.; Recher, C.; Dusanter-Fourt, I.; et al. CXXC5 (retinoid-inducible nuclear factor, RINF) is a potential therapeutic target in high-risk human acute myeloid leukemia. *Oncotarget* **2013**, *4*, 1438–1448, doi:10.18632/oncotarget.1195.
9. Bruserud, Ø.; Reikvam, H.; Fredly, H.; Skavland, J.; Hagen, K.M.; van Hoang, T.T.; Brenner, A.K.; Kadi, A.; Astori, A.; Gjertsen, B.T.; et al. Expression of the potential therapeutic target CXXC5 in primary acute myeloid leukemia cells—High expression is associated with adverse prognosis as well as altered intracellular signaling and transcriptional regulation. *Oncotarget* **2015**, *6*, 2794–2811, doi:10.18632/oncotarget.3056.
10. Kühnl, A.; Valk, P.J.; Sanders, M.A.; Ivey, A.; Hills, R.K.; Mills, K.I.; Gale, R.E.; Kaiser, M.F.; Dillon, R.; Joannides, M.; et al. Downregulation of the Wnt inhibitor CXXC5 predicts a better prognosis in acute myeloid leukemia. *Blood* **2015**, *125*, 2985–2994, doi:10.1182/blood-2014-12-613703.
11. Lee, J.W.; Kim, H.S.; Hwang, J.; Kim, Y.H.; Lim, G.Y.; Sohn, W.J.; Yoon, S.R.; Kim, J.Y.; Park, T.S.; Oh, S.H.; et al. Regulation of HOXA9 activity by predominant expression of DACH1 against C/EBPalpha and GATA-1 in myeloid leukemia with MLL-AF9. *Biochem. Biophys. Res. Commun.* **2012**, *426*, 299–305, doi:10.1016/j.bbrc.2012.08.048.
12. Zhu, Q.; Zhang, L.; Li, X.; Chen, F.; Jiang, L.; Yu, G.; Wang, Z.; Yin, C.; Jiang, X.; Zhong, Q.; et al. Higher EZH2 expression is associated with extramedullary infiltration in acute myeloid leukemia. *Tumour. Biol.* **2016**, *37*, 11409–11420, doi:10.1007/s13277-016-4983-4.
13. Bosman, M.C.; Schepers, H.; Jaques, J.; Brouwers-Vos, A.Z.; Quax, W.J.; Schuringa, J.J.; Vellenga, E. The TAK1-NF-kappaB axis as therapeutic target for AML. *Blood* **2014**, *124*, 3130–3140, doi:10.1182/blood-2014-04-569780.
14. Xie, X.; Wang, X.; Liao, W.; Fei, R.; Wu, N.; Cong, X.; Chen, Q.; Wei, L.; Wang, Y.; Chen, H. MYL6B, a myosin light chain, promotes MDM2-mediated p53 degradation and drives HCC development. *J. Exp. Clin. Cancer Res.* **2018**, *37*, 28, doi:10.1186/s13046-018-0693-7.
15. Chen, J.; Li, C.; Zhan, R.; Yin, Y. SPG6 supports development of acute myeloid leukemia by regulating BMPR2-Smad-Bcl-2/Bcl-xl signaling. *Biochem. Biophys. Res. Commun.* **2018**, *501*, 220–225, doi:10.1016/j.bbrc.2018.04.220.
16. D'Arcy, P.; Wang, X.; Linder, S. Deubiquitinase inhibition as a cancer therapeutic strategy. *Pharmacol. Ther.* **2015**, *147*, 32–54, doi:10.1016/j.pharmthera.2014.11.002.
17. Bera, R.; Chiu, M.C.; Huang, Y.J.; Liang, D.C.; Lee, Y.S.; Shih, L.Y. Genetic and Epigenetic Perturbations by DNMT3A-R882 Mutants Impaired Apoptosis through Augmentation of PRDX2 in Myeloid Leukemia Cells. *Neoplasia* **2018**, *20*, 1106–1120, doi:10.1016/j.neo.2018.08.013.
18. Jin, Y.; Yang, Q.; Liang, L.; Ding, L.; Liang, Y.; Zhang, D.; Wu, B.; Yang, T.; Liu, H.; Huang, T.; et al. Compound kushen injection suppresses human acute myeloid leukaemia by regulating the Prdxs/ROS/Trx1 signalling pathway. *J. Exp. Clin. Cancer Res.* **2018**, *37*, 277, doi:10.1186/s13046-018-0948-3.
19. Upadhyay, G.; Chowdhury, A.H.; Vaidyanathan, B.; Kim, D.; Saleque, S. Antagonistic actions of Rcor proteins regulate LSD1 activity and cellular differentiation. *Proc. Natl. Acad. Sci. USA* **2014**, *111*, 8071–8076, doi:10.1073/pnas.1404292111.
20. Hoshii, T.; Cifani, P.; Feng, Z.; Huang, C.H.; Koche, R.; Chen, C.W.; Delaney, C.D.; Lowe, S.W.; Kentsis, A.; Armstrong, S.A. A Non-catalytic Function of SETD1A Regulates Cyclin K and the DNA Damage Response. *Cell* **2018**, *172*, 1007–1021.e1017, doi:10.1016/j.cell.2018.01.032.

21. Buchwald, M.; Pietschmann, K.; Muller, J.P.; Bohmer, F.D.; Heinzel, T.; Kramer, O.H. Ubiquitin conjugase UBCH8 targets active FMS-like tyrosine kinase 3 for proteasomal degradation. *Leukemia* **2010**, *24*, 1412–1421, doi:10.1038/leu.2010.114.
22. Lin, X.C.; Xu, Y.; Sun, G.P.; Wen, J.L.; Li, N.; Zhang, Y.M.; Yang, Z.G.; Zhang, H.T.; Dai, Y. Molecular dysfunctions in acute myeloid leukemia revealed by integrated analysis of microRNA and transcription factor. *Int. J. Oncol.* **2016**, *48*, 2367–2380, doi:10.3892/ijo.2016.3489.
23. Torrebadell, M.; Diaz-Beya, M.; Kalko, S.G.; Pratcorona, M.; Nomdedeu, J.; Navarro, A.; Gel, B.; Brunet, S.; Sierra, J.; Camos, M.; et al. A 4-gene expression prognostic signature might guide post-remission therapy in patients with intermediate-risk cytogenetic acute myeloid leukemia. *Leuk. Lymphoma.* **2018**, *59*, 2394–2404, doi:10.1080/10428194.2017.1422859.
24. Kwon, H.Y.; Bajaj, J.; Ito, T.; Blevins, A.; Konuma, T.; Weeks, J.; Lytle, N.K.; Koechlein, C.S.; Rizzieri, D.; Chuah, C.; et al. Tetraspanin 3 Is Required for the Development and Propagation of Acute Myelogenous Leukemia. *Cell Stem Cell* **2015**, *17*, 152–164, doi:10.1016/j.stem.2015.06.006.
25. Maeda, T. Regulation of hematopoietic development by ZBTB transcription factors. *Int. J. Hematol.* **2016**, *104*, 310–323, doi:10.1007/s12185-016-2035-x.
26. Honnemyr, M.; Bruserud, Ø.; Brenner, A.K. The constitutive protease release by primary human acute myeloid leukemia cells. *J. Cancer Res. Clin. Oncol.* **2017**, *143*, 1985–1998, doi:10.1007/s00432-017-2458-7.
27. Sun, X.; Huang, S.; Wang, X.; Zhang, X.; Wang, X. CD300A promotes tumor progression by PECAM1, ADCY7 and AKT pathway in acute myeloid leukemia. *Oncotarget* **2018**, *9*, 27574–27584, doi:10.18632/oncotarget.24164.
28. Fang, J.; Ying, H.; Mao, T.; Fang, Y.; Lu, Y.; Wang, H.; Zang, I.; Wang, Z.; Lin, Y.; Zhao, M.; et al. Upregulation of CD11b and CD86 through LSD1 inhibition promotes myeloid differentiation and suppresses cell proliferation in human monocytic leukemia cells. *Oncotarget* **2017**, *8*, 85085–85101, doi:10.18632/oncotarget.18564.
29. Bertoli, S.; Paubelle, E.; Berard, E.; Saland, E.; Thomas, X.; Tavitian, S.; Larcher, M.V.; Vergez, F.; Delabesse, E.; Sarry, A.; et al. Ferritin heavy/light chain (FTH1/FTL) expression, serum ferritin levels, and their functional as well as prognostic roles in acute myeloid leukemia. *Eur. J. Haematol.* **2019**, *102*, 131–142, doi:10.1111/ejh.13183.
30. Kikushige, Y.; Miyamoto, T. Identification of TIM-3 as a Leukemic Stem Cell Surface Molecule in Primary Acute Myeloid Leukemia. *Oncology* **2015**, *89 Suppl 1*, 28-32, doi:10.1159/000431062.
31. Xu, L.; Xu, J.; Ma, S.; Li, X.; Zhu, M.; Chen, S.; Han, Y.; Tang, X.; Fu, Z.; Qiu, H.; et al. High Tim-3 expression on AML blasts could enhance chemotherapy sensitivity. *Oncotarget* **2017**, *8*, 102088–102096, doi:10.18632/oncotarget.22141.
32. Alasseiri, M.; Ahmed, A.U.; Williams, B.R.G. Mechanisms and consequences of constitutive activation of integrin-linked kinase in acute myeloid leukemia. *Cytokine Growth Factor Rev.* **2018**, *43*, 1–7, doi:10.1016/j.cytogfr.2018.06.001.
33. de la Puente, P.; Weisberg, E.; Muz, B.; Nonami, A.; Luderer, M.; Stone, R.M.; Melo, J.V.; Griffin, J.D.; Azab, A.K. Identification of ILK as a novel therapeutic target for acute and chronic myeloid leukemia. *Leuk Res.* **2015**, 10.1016/j.leukres.2015.09.005, doi:10.1016/j.leukres.2015.09.005.
34. Handschuh, L.; Kazmierczak, M.; Milewski, M.C.; Goralski, M.; Luczak, M.; Wojtaszewska, M.; Uszczynska-Ratajczak, B.; Lewandowski, K.; Komarnicki, M.; Figlerowicz, M. Gene expression profiling of acute myeloid leukemia samples from adult patients with AML-M1 and -M2 through boutique microarrays, real-time PCR and droplet digital PCR. *Int. J. Oncol.* **2018**, *52*, 656–678, doi:10.3892/ijo.2017.4233.
35. Fang, Y.; Xie, L.N.; Liu, X.M.; Yu, Z.; Kong, F.S.; Song, N.X.; Zhou, F. Dysregulated module approach identifies disrupted genes and pathways associated with acute myelocytic leukemia. *Eur. Rev. Med. Pharmacol. Sci* **2015**, *19*, 4811–4826.
36. Höckendorf, U.; Yabal, M.; Jost, P.J. RIPK3-dependent cell death and inflammasome activation in FLT3-ITD expressing LICs. *Oncotarget* **2016**, *7*, 57483–57484, doi:10.18632/oncotarget.11195.
37. Eisele, L.; Klein-Hitpass, L.; Chatzimanolis, N.; Opalka, B.; Boes, T.; Seeber, S.; Moritz, T.; Flasshove, M. Differential expression of drug-resistance-related genes between sensitive and resistant blasts in acute myeloid leukemia. *Acta. Haematol.* **2007**, *117*, 8–15, doi:10.1159/000096854.

38. Kim, D.M.; Choi, S.H.; Yeom, Y.I.; Min, S.H.; Kim, I.C. Genome-scale functional analysis of the human genes modulating p53 activity by regulating MDM2 expression in a p53-independent manner. *Biochem. Biophys. Res. Commun.* **2016**, *478*, 976–981, doi:10.1016/j.bbrc.2016.08.063.
39. Villamón, E.; González-Fernández, J.; Such, E.; Cervera, J.V.; Gozalbo, D.; Luisa Gil, M. Imiquimod inhibits growth and induces differentiation of myeloid leukemia cell lines. *Cancer Cell Int.* **2018**, *18*, 15, doi:10.1186/s12935-018-0515-1.

© 2019 by the authors. Licensee MDPI, Basel, Switzerland. This article is an open access article distributed under the terms and conditions of the Creative Commons Attribution (CC BY) license (http://creativecommons.org/licenses/by/4.0/).
